# RGD-Binding Integrins in Prostate Cancer: Expression Patterns and Therapeutic Prospects against Bone Metastasis

**DOI:** 10.3390/cancers4041106

**Published:** 2012-10-26

**Authors:** Mark Sutherland, Andrew Gordon, Steven D. Shnyder, Laurence H. Patterson, Helen M. Sheldrake

**Affiliations:** Institute of Cancer Therapeutics, University of Bradford, Bradford, BD7 1RL, UK; E-Mails: m.sutherland@bradford.ac.uk (M.S.); 325611@gmail.com (A.G.); s.d.shnyder@bradford.ac.uk (S.D.S.); l.h.patterson@bradford.ac.uk (L.H.P.)

**Keywords:** integrin, RGD, prostate carcinoma, bone metastasis

## Abstract

Prostate cancer is the third leading cause of male cancer deaths in the developed world. The current lack of highly specific detection methods and efficient therapeutic agents for advanced disease have been identified as problems requiring further research. The integrins play a vital role in the cross-talk between the cell and extracellular matrix, enhancing the growth, migration, invasion and metastasis of cancer cells. Progression and metastasis of prostate adenocarcinoma is strongly associated with changes in integrin expression, notably abnormal expression and activation of the β_3_ integrins in tumour cells, which promotes haematogenous spread and tumour growth in bone. As such, influencing integrin cell expression and function using targeted therapeutics represents a potential treatment for bone metastasis, the most common and debilitating complication of advanced prostate cancer. In this review, we highlight the multiple ways in which RGD-binding integrins contribute to prostate cancer progression and metastasis, and identify the rationale for development of multi-integrin antagonists targeting the RGD-binding subfamily as molecularly targeted agents for its treatment.

## Abbreviations

Akt: protein kinase B
BBN: bombesin
BSP: bone sialoprotein
DOTA: 1,4,7,10-tetraazacyclododecane-1,4,7,10-tetraacetic acid
DSPP: dental sialophosphoprotein
ECM: extracellular matrix
EGFR: epidermal growth factor receptor
EMT: epithelial mesenchymal transition
ERK: extracellular signal-regulated kinase
FACS: fluorescence activated cell sorting
FAK: focal adhesion kinase
Fg: fibrinogen
Fn: fibronectin
GRPR: gastrin releasing peptide receptor
Hsp90: heat shock protein 90
HUVEC: human umbilical vein endothelial cell
IGF-1R: insulin-like growth factor type 1 receptor
LAP: latency associated peptide
MEK: mitogen-activated protein kinase/extracellular signal-regulated kinase kinase
MMP: matrix metalloproteinase
MRI: magnetic resonance imaging
NODA: 1,4,7-triazacyclononane-1,4-diacetic acid
NOTA: 1,4,7-triazacyclononane-1,4,7-triacetic acid
PET: positron emission tomography
PI3K: phosphoinositide 3-kinase
PSA: prostate specific antigen
OPN: osteopontin
SIBLING: small integrin binding *N*-linked glycoprotein
SMART: Simultaneously Multiple Aptamers and RGD Targeting
SPARC: secreted protein acidic and rich in cysteine
TCIPA: tumour cell-induced platelet aggregation
TGF-β: transforming growth factor-β
TNF-α: tumour necrosis factor-α
VEGF(R): vascular endothelial growth factor (receptor)
Vn: vitronectin
vWF: von Willebrand factor

Standard 1 letter codes for amino acids are used throughout.

## 1. Introduction

Prostate cancer is the most commonly occurring cancer in men in the developed world, and the second most common worldwide, with over 900,000 new cases and 250,000 deaths estimated to occur annually [1]. Survival rates are increasing, largely due to improvements in detection and treatment of early stage disease, however, the treatment of advanced prostate cancer remains a challenge. As the disease progresses, tumours become castration resistant and no longer respond to hormonal deprivation therapies [2]. Bone metastases are a common occurrence in castration-resistant prostate cancer, affecting up to 80% of patients, and result in significantly reduced quality of life through pain and pathological fractures [3]. Metastases to other sites, such as distal lymph nodes and lung, are also features of advanced disease. There are currently few effective therapeutic agents for advanced prostate cancer, with the most frequently used agent, docetaxel, often poorly tolerated [2]. Although a number of new drugs such as abiretarone acetate and alpharadin are progressing into the clinic [4], there remains a need to identify molecularly targeted agents to control tumour dissemination for survival benefit. Furthermore, discovery of biomarkers of disease progression will provide new diagnostics for early detection and prognostic indicators for treatment outcome.

The integrin family of cell surface glycoproteins control cell-extracellular matrix (ECM) adhesion and signalling across the cell membrane. Integrins are heterodimers made up of an α and a β glycoprotein subunit, each comprising a multidomain extracellular section which interacts with extracellular proteins, a single transmembrane domain, and a short cytoplasmic tail which interacts with intracellular signalling effectors and kinases [5,6].

Integrins can change conformation in response to intracellular signals or extracellular ligand binding; the active conformation is characterised by extension of the receptor headpiece above the cell surface, increased affinity for ligands, and increased integrin-mediated signalling [7,8,9]. A useful pictorial summary of integrins and their ligands has been published by Humphries [10].

Integrins may be divided into classes according to cellular expression, subunit identity or ligands bound [11,12]. The RGD-recognising integrins are a family of 8 integrins (Table 1) which bind the common Arg-Gly-Asp tripeptide sequence at a binding site formed at the junction of the α and β subunit headpieces. α_5_β_1_ and α_8_β_1_ possess synergy sites in addition to the RGD binding site, where the binding of PHSRN and LFEIFEIER sequences increases ligand integrin affinity. Considerable detail regarding ligand binding is available for integrins that have become the focus for drug design: crystal structures have been determined for α_IIb_β_3_ [13], α_v_β_3_ [14,15,16] and α_5_β_1_ [17], and homology models for α_v_β_5_ [18] and α_5_β_1_ [19].

**Table 1:** RGD-binding integrins [20,21,22,23,24,25,26].

Integrin	Common Ligands	Major physiological roles
α_v_β_1_	Fibrinogen (Fg), Fibronectin (Fn), Vitronectin (Vn), Osteopontin (OPN), LAP-TGF-β	
α_v_β_3_	Fg, Von Willebrand factor (vWF), Fn, Vn, Bone sialoprotein (BSP), OPN, LAP-TGF-β	Angiogenesis Bone resorption
α_v_β_5_	Fn, Vn, BSP, LAP-TGF-β	Angiogenesis Vascular permeability
α_v_β_6_	Fn, Vn, OPN, LAP-TGF-β	Lung development and physiology TGF-β activation
α_v_β_8_	Vn, LAP-TGF-β	TGF-β activation Angiogenesis Brain development
α_5_β_1_	Fn, OPN	Angiogenesis
α_8_β_1_	Fn, Vn, Tenascin, OPN, LAP-TGF-β, Nephronectin	Kidney and lung development Hair cell differentiation and function
α_IIb_β_3_	Fg, Fn, Vn, vWF	Platelet aggregation

A number of the RGD-recognising integrins, α_v_β_3_, α_v_β_5_, α_v_β_8_ and α_5_β_1_, play important roles in controlling angiogenesis [27,28]. α_v_β_3_ is directly involved in bone turnover through mediating osteoclast adhesion and function [29] and thus is identified as a drug target for treatment of osteoporosis and bone metastasis [30,31]. α_v_β_3_ expression on other cell types, and other integrins, including α_v_β_5_ and α_5_β_1_ expressed on cancer and endothelial cells, and platelet α_IIb_β_3_ [31] also contribute to the process of bone metastasis, facilitating tumour cell survival in the blood stream, migration, and adhesion at the metastatic site [31,32,33,34].

Previous reviews have covered detailed aspects of integrin expression and signalling in cancer [35], including the non-RGD binding integrins such as α_2_β_1_, α_6_β_1_ and α_6_β_4_ in prostate cancer [32,36,37,38]. Here, we summarise key findings on the expression and function of the RGD-binding integrin subfamily in prostate cancer, and review drugs and imaging agents currently in development that target these receptors.

## 2. Expression of RGD-Binding Integrins in Prostate Cancer

### 2.1. Clinical Prostate Adenocarcinoma

The expression of RGD-binding integrins in clinical tissue samples is summarised in Table 2. Primary cells isolated from tissue samples of prostate adenocarcinoma expressed high levels of functional α_v_β_3_, in contrast to epithelial cells from normal tissue which are α_v_β_3_ negative. FACS analysis for α_v_, α_5_ and β_1_ integrin subunits showed little difference in expression between cells from tumour and normal tissue, suggesting α_v_β_3_ is responsible for tumour cell migration towards and adhesion to vitronectin [39]. The expression of the α_v_ subunit in primary cultures has subsequently been shown to be highly variable (0.1–51.9% of cells), and indicative of the tumour stem cell population in a particular sample [40].

**Table 2:** Summary of RGD-binding integrin expression observed in prostate adenocarcinoma.

Integrin	Expressed in normal prostate tissue?	Expressed in primary prostate tumours?	Expressed in metastases?	Reference
α_v_β_1_	Yes			[41]
α_v_β_3_	No	Yes	Yes	[39,42,43,44,45,46]
α_v_β_5_	No	Yes	Yes	[45,47]
α_v_β_6_	No	Yes		[47,48]
α_v_β_8_	No	No		[47]
α_5_β_1_	Yes	Reduced	Yes	[44,49]
α_IIb_β_3_	No	Yes	Yes	[42,50]

Prostate cancer is one of a small number of tumours that ectopically express α_IIb_β_3_, an integrin originally believed to be found only on platelets. α_IIb_ mRNA was originally detected in human tumour samples by *in situ* hybridisation [50]; α_IIb_ protein expression has since been confirmed by other techniques [42].

Truncated forms of integrin subunits have also been found in tumour samples. Truncated variants of both α_IIb_ [51] and β_3_ [52] have been detected in tumour samples with intermediate or advanced Gleason grade. Both truncated forms are also expressed in DU145 and PC-3 prostate cancer cell lines, and have been shown to be secreted by the cells and prevent their adhesion to integrin ligands. It is interesting to speculate that expression of truncated integrins could facilitate tumour migration by diminishing ECM adherence.

An analysis of the association between tumour integrin expression and the likelihood of biochemical recurrence after surgical removal of an apparently localised tumour found the majority of 111 prostate tumours expressed α_v_, α_v_β_3_ and α_IIb_β_3_ integrins [42]. The pattern of α_v_ and α_v_β_3_ expression was the same in recurrent and non-recurrent tumours; 25–28% of each group showed no expression, while the majority were classified as moderate or high expressing. Over 90% of tumours were α_IIb_β_3_-positive. α_IIb_β_3_ expression was stronger in recurrent tumours (40% strongly expressing compared to 20% of non-recurrent tumours), and was identified as marginally significant for recurrence, whereas high expression of α_3_β_1_ was highly significant as a prognostic indicator.

In contrast, a comparison of paired samples of primary prostate tumours and lymph node metastasis from 19 patients found “abnormal” expression of α_v_ and α_v_β_3_ in all cases. Expression was classified as abnormal if immunohistochemical staining was negative, weak, moderate or focal. Metastasis was frequently associated with a decrease in integrin expression, with α_v_ expression increasing in 6% of cases and decreasing in 59%, and α_v_β_3_ expression decreasing in 47% of cases [43]. These results should be interpreted with caution since strong expression of α_v_β_3_ only occurs normally on activated endothelial cells. Weak or moderate ectopic expression of a functional integrin could be highly significant for cell proliferation and spreading. An observational cohort study on 64,545 men provided 1,172 cases of prostate cancer where samples could be analysed to determine molecular markers of aggressive disease. Unfortunately, β_3_ integrin expression could not be detected by immunohistochemical analysis in the archival tumour samples [53].

Normal prostate tissue has been reported to express α_v_β_1_ but not α_5_β_1_ [41]. In 20 cases of primary prostate cancer, one expressed α_5_β_1_; the expression of α_v_ and other β subunits was not reported [41]. Expression of the α_5_ and β_1_ subunits has been shown to be negatively correlated with clinical tumour grade, with a comparison of 30 primary prostate tumours and 30 normal prostate samples showing a significant reduction of α_5_ and β_1_ expression in the tumour samples [49]. In contrast, a comparison of biopsy samples from benign prostatic hypertrophy and primary prostate tumours found β_1_ expression increased with tumour grade and became located on the surface of tumour cells. Low levels of β_1_ expression were observed in areas of benign disease, although these samples included apparently normal biopsies from patients with diagnosed prostate cancer [54]. Weak β_3_ expression was also present in 25% of tumour areas.

A meta-analysis of genes involved in prostate cancer progression noted a general trend for downregulation of integrins (both RGD and non-RGD binding) and their ligands (notably, changing the expression pattern of collagens) during cancer progression [55]. *ITGAV* and *ITGB5* were upregulated in prostatic intraepithelial neoplasia compared to normal prostate, and in the transition from normal prostate to non-metastatic cancer *ITGA5*, *B1*, *B3* and *B6* were downregulated, and *ITGA2B*, *AV* and *B5* were upregulated. Expression of *ITGA5* was negatively correlated with Gleason score. It has been proposed that the decrease in integrin expression allows cells to escape integrin-mediated cell death resulting from decreased collagen expression as part of the normal aging process, and leads to increased cell motility and proliferation driving tumourigenesis. However, further work is required to correlate this analysis of integrin message with integrin protein expression.

Screening a tissue microarray of archival human tumours using a panel of antibodies to α_v_ subfamily integrins showed prostate cancer samples strongly expressed α_v_ and α_v_β_5_ in the tumour cells and stroma, with some expression of α_v_β_3_ on the tumour vasculature. Small regions of tumour were positive for α_v_β_6_, and there was no expression of α_v_β_8_ [47].

Studies on the expression of α_v_β_6_ show similar contradictory results to those on α_v_β_3._ Immunohistochemical analysis of 40 primary prostate tumours found 77% were α_v_β_6_ negative, but high integrin expression was observed in basal cells in ducts with high-grade intraepithelial neoplasia and in normal tissue adjacent to tumours [48]. Other studies have shown that α_v_β_6_ is expressed in epithelial cells in prostate tumours, and focally in areas of inflammation, proliferative inflammatory atrophy, and prostate intraepithelial neoplasia [56]. Similar expression patterns were observed in transgenic mouse models of prostate disease. In the Pten^pc−/−^ model of spontaneous prostate cancer, α_v_β_6_ was expressed on malignant epithelial cells in prostatic intraepithelial neoplasia and invasive adenocarcinoma. α_v_β_6_ expression was also induced in the POET model of prostate inflammation. α_v_β_6_ is proposed to support metastasis by activating TGF-β1, which is associated with metastasis and poor prognosis [57].

Characteristic patterns of integrin expression have been found present in bone metastasis. Tumour cells obtained from the bone marrow of patients without overt metastases expressed α_5_, α_v_, β_1_ and β_3_ integrin subunits, a pattern which appeared to be characteristic of micrometastatic cells, and which was hypothesised to promote homing and survival in bone marrow [44]. These integrins may therefore represent potential targets in preventing bone colonisation. α_v_β_3_ and α_v_β_5_ have been found to be expressed in the active conformation on prostate tumours; comparison of matched samples of primary tumour and bone metastasis showed that integrin activation is increased in bone metastasis compared to the primary tumour [45]. Increased expression of the β_3_ subunit has been found in prostate cancer orbital metastases [46]. β_3_ expression is hypothesised to be responsible for the tendency of prostate cancer to metastasise to bone, but despite substantial evidence for this role in preclinical models, and demonstrated efficacy of integrin antagonists in treating bone metastasis (*vide infra*) there have been no large studies on integrin expression in bone metastases.

### 2.2. Preclinical Models of Prostate Cancer

Compared to other types of cancer, a relatively small number of cell line models are commonly used to study prostate adenocarcinoma.Reports of the expression of RGD-binding integrins in these commonly used prostate carcinoma cell line models are summarised in Table 3 and Table 4.

**Table 3:** RGD-binding integrin heterodimer expression on the surface of prostate cancer cell lines. Key: +++ very high; ++ high; + moderate; +/− low; − negative; blank not tested; ns expressed but level not stated.

Cell line	α_IIb_β_3_	α_v_β_3_	α_v_β_1_	α_v_β_5_	α_5_β_1_	α_v_β_6_	α_v_β_8_	Reference
PC-3	−	+	+++	++				[58]
PC-3		−		++	++			[59]
PC-3		−		++		+/−	+	[47]
PC-3	+							[34,50]
PC-3		++						[60]
PC-3					++			[61]
PC-3		++		++	++	++		[62]
PC-3		–						[63]
PC-3						++		[57]
PC-3		+++						[64]
PC-3		+++			+++			[65]
DU145		+/–		++		−	−	[47]
DU145	+							[34,50]
DU145		++						[66]
DU145					++			[61]
DU145						++		[48]
DU145		+						[63]
LNCaP		++						[60]
LNCaP					++			[61]
LNCaP		−		+	ns	+/−		[39]
LNCaP		++						[63]
LNCaP		++						[64]
C4-2		++						[60]
C4-2		++						[64]

**Table 4:** RGD-binding integrin subunit expression on the surface of prostate cancer cell lines. Key: +++ very high; ++ high; + moderate; +/− low; − negative; blank not tested; ns expressed but level not stated.

Cell line	α_IIb_	α_v_	α_5_	β_1_	β_3_	β_5_	β_6_	β_8_	Reference
PC-3		+++		+++	+++	+	−		[39]
PC-3		+	++	+++	+	+/−			[67]
PC-3			+/−	ns					[68]
PC-3	−	+	+	+++		+/−			[69]
PC-3		+++			+++				[64]
PC-3			+	++	+				[70]
DU145		+	++	+++	+/−	+/−			[67]
DU145			+		+				[71]
DU145	−	+	++	++	+/−	+/−			[69]
LNCaP		++		+++	−	+	+/−		[39]
LNCaP		++		++	+				[72]
LNCaP		++			−				[64]
LNCaP			+	+	−				[70]
C4-2		+++							[40]
C4-2		++		++	+				[72]
C4-2		+++			−				[64]
DuPro1	−	+	+	++	−	+			[69]

It is noteworthy that contrasting patterns of expression are frequently observed between cell lines used in different laboratories; for example, the expression of α_v_β_3_ in both PC-3 and LNCaP cells ranges from high to absent, an observation that has been attributed to response to differences in cell culture conditions, or the development of clonal variations or cell line heterogeneity in long term culture [63,65]. The variability of expression of α_v_β_3_ and α_v_β_6_ in cell lines parallels that seen in tumour samples whereas α_v_β_5_ and α_5_β_1_ are generally always expressed in cell lines. α_v_β_6_, and α_v_β_8_ expression is less well-investigated compared to other integrins, and there are no reported studies on α_8_β_1_.

To improve understanding and reproducibility of models of prostate cancer, changes in integrin expression in response to the environment in which a cell line is grown have been investigated. In cells grown in culture, the expression of all integrins is significantly downregulated on the surface of highly confluent cells [69]. α_IIb_β_3_ surface expression was observed to be higher in orthotopically implanted DU145 tumours than in subcutaneous xenografts; ectopic expression of α_IIb_β_3_ decreased when the cells were cultured [34]. α_v_ gene expression was upregulated in PC-3 cells grown in contact with normal prostate stromal cells, and downregulated in contact with long bone osteoblasts [73]. Expression of α_v_ and β_3_ mRNA measured by quantitative PCR was correlated with tumourigenicity in LNCaP, C4-2 and PC-3 cell lines grown in culture and as xenografts, although α_v_β_3_ protein levels were similar in all cell lines and lowest in PC-3 [60]. Changes in integrin mRNA levels when cell lines were grown as xenografts were cell line dependent; expression increased in the more tumourigenic cell lines, with a very large increase in β_3_ levels observed in PC-3 xenografts [60]. Similarly, exposure of LNCaP to dehydrotestosterone increased the expression of α_v_ and VEGF mRNA, but had no effect on PC-3 [74]. *In vivo*, factors in the local microenvironment, which are not normally present in cell culture media, can control integrin expression. For example, intratesticular inoculation increased α_v_ mRNA expression in both LNCaP and PC-3 xenografts, and resulted in increased tumour growth and metastasis compared to orthotopic implantation of LNCaP, due to enhanced angiogenesis and interaction with the tumour stroma [74].

Integrin receptor expression can change over time, due to tumour progression and intratumour heterogeneity in *in vivo* models and clinical cases, and due to development of clonal variations and adaptation to the levels of protein ligands available when cell lines are grown in culture. Additionally, integrin expression in xenografts can change in response to the tumour environment and thus differ markedly from the same tumour model *in vitro*. It is therefore important that tumour models used to investigate integrin biology or integrin-targeted drugs be fully characterised to ensure they retain the receptors of interest in a particular situation, for example when cultured in specific media/cell density or grown as xenografts. Most current studies do not assess the full integrin profile of the cell lines used; this is an important consideration when assessing the biological response to antagonists with different receptor selectivity profiles, given the ability of multiple integrins to recognise the same ligands, control the same physiological processes, and interact with one another through trans-dominant inhibition.

## 3. Consequences of Integrin Expression in Prostate Cancer

### 3.1. α_v_ Subfamily Integrins

The most thoroughly investigated α_v_ subfamily heterodimer, α_v_β_3_, is overexpressed on activated endothelial cells and in a wide range of cancer types [75]. Its role in prostate cancer was previously reviewed by Cooper *et al.* in 2002 [76]. α_v_β_3_ expression and abnormal activation in cancer is commonly associated with disease progression and poor prognosis [75]. As cell lines develop a more metastatic phenotype, integrin use changes and α_v_β_3_ expression increases; C4-2 requires α_v_β_3_ for adhesion and migration, whereas the parent LNCaP cell line used α_v_β_5_ for adhesion and was unable to migrate on OPN [72]. Transformation of LNCaP to an androgen-independent model of prostate cancer, LNCaP-19 resulted in increased cell proliferation, migration, *in vivo* invasive ability, and adhesion to vitronectin and fibronectin. However the expression of α_v_, β_3_, α_5_ and β_1_ was unchanged, suggesting that increased integrin activation may be responsible for the observed increase in cells’ adhesive properties [77].

α_v_β_3_ expression allows cells to adhere to and migrate on Vn [39,67,78], Fn [39,67] and OPN [78]. Binding to Vn activates signalling through FAK [39] and PI3K/Akt pathways; activation of PI3K/Akt signalling is specifically required for adhesion and migration on Vn to take place [78] and Akt1 activity has been shown to promote adhesion, migration on ECM ligands and transendothelial migration by regulating β_3_ integrin activation [79].

α_v_β_3_ and α_5_β_1_ mediate prostate cancer cell interactions with endothelial cells through adhesion to Vn and Fn on the endothelial cell surface and in their surroundings [67]. β_3_ integrin in endothelial cells are associated with focal contacts—regions of firm adhesion—whereas in PC-3 cells, β_3_ is located in pseudopodia and membrane ruffles associated with cell motility. Use of antisense oligonucleotides for β_3_ has shown that functional β_3_ is required for the later stages of transendothelial migration; it enables cells to extend membrane protrusions and interact with the underlying ECM proteins [80]. 

Binding to Vn has been recently demonstrated to induce differentiation of prostate cancer stem cells; α_v_β_3_ is the most important receptor controlling differentiation, with α_v_β_5_ making a smaller contribution. Antagonism of α_v_β_3_ with anti-α_v_β_3_ antibodies or the cyclic peptide cRGDfK prevented stem cell differentiation, activation of FAK signalling, and development of tumours *in vivo* following injection of stem cells [81]. The discovery that α_v_β_3_-engagement and signalling is necessary for tumourigenesis is an important one: maintaining stem cells in a dormant state by use of integrin antagonists represents a new strategy for targeted therapy.

Crude extract of proteins from mineralised bone supported adhesion of DU145 cells which was blocked by RGD peptide or anti-α_v_β_3_ antibody [82]. RGD-containing integrin ligands found at high levels in the bone microenvironment include Fn, Vn, OPN, and other members of the small integrin-binding ligand *N*-linked glycoprotein (SIBLING) family of proteins, namely BSP, dentin matrix protein 1, dentin sialophosphoprotein (DSPP), and matrix extracellular phosphoglycoprotein [83]. Expression of SIBLINGS, notably BSP and OPN is associated with prostate cancer progression [84,85,86,87]; OPN [86] and BSP [84] can be used as biomarkers of bone metastasis and DSPP has been proposed as a biomarker for prostate cancer diagnosis [88].

Osteopontin is a chemoattractant for prostate cancer cells, potentially promoting preferential formation of bone tumours [89]. OPN increases the metastatic capability of a cell by increasing the secretion of plasminogen activators [89] and MMP-9 [90] and the formation of invadopodia (cell membrane structures used in adhesion and migration) [91], all through α_v_β_3_ signalling pathways. α_v_-integrin mediated binding of prostate epithelial cells to OPN stimulated the growth of a subpopulation of cells with high proliferative potential, possibly stem cells [92]. Subsequently, the a_v_ subunit has been shown to be a marker of prostate cancer stem cells [40]. Bone sialoprotein has also been shown to increase expression of MMPs, promote integrin-mediated migration, and upregulate integrin survival signalling pathways in prostate carcinoma cells. Transfection of PC-3 cells with BSP increased expression of α_v_, β_3_ and β_5_, formation of focal adhesions, and integrin-mediated migration and survival signalling; blocking integrin-BSP interaction and the resulting signalling through α_v_β_3_/α_v_β_5_ could provide a new anti-metastatic strategy [93].

A number of proteins have been implicated as driving factors for bone-specific metastasis. α_v_β_3_ and α_v_β_5_ mediate the preferential migration of metastatic prostate cancer cells to bone-derived secreted protein acidic and rich in cysteine (SPARC). Tumour growth and migration are supported by an autocrine loop, where SPARC binding to α_v_β_5_ results in increased levels of VEGF and VEGFR2, which in turn activate α_v_β_3_ and α_v_β_5_ and stimulate cell proliferation. Upregulation of VEGF production by SPARC provides metastatic prostate cancer cells with a specific growth advantage in the bone microenvironment, thus blocking the interaction of SPARC with activated α_v_ integrins should reduce bone metastasis [45]. Studies with isogenic prostate cancer cell lines expressing α_v_β_3_ in a range of activation states have further shown that functionally active α_v_β_3_ is required for the growth of bone metastasis. α_v_β_3_-negative tumour cells promote bone loss, but α_v_β_3_ is required for bone deposition and an osteoblastic phenotype. Locking α_v_β_3_ in an inactive or active conformation reduced tumour growth; inactive α_v_β_3_ is unable to support cell adhesion, whereas permanently active integrin supports strong adhesion but cannot regulate adhesion or signalling [94].

Exposure of bone marrow mesenchymal stems cells to conditioned medium from bone metastatic prostate cancer cells upregulated expression of α_5_ and α_v_ mRNA more than 8-fold; β_1_ and β_3_ expression was increased by a lesser extent. Expression of the integrin ligand Fn was also significantly increased. These changes promote differentiation into osteoblasts, initiating a mechanism for formation of new bone once metastasis has become established in the bone [95]. Exposure of metastatic prostate cancer cells to stromal cell-derived factor 1 increased the expression of high affinity (activated) α_v_β_3_, promoting adhesion to bone marrow endothelial cells, VN, and OPN. Stromal cell-derived factor 1 is present at high levels in the bone microenvironment, therefore may promote integrin-mediated adhesion, invasion, and proliferation of metastatic cells once they reach the bone marrow [64]. Bone morphogenetic protein-2 secreted by osteoblasts induced migration in both androgen dependent and androgen independent prostate cancer cell lines by upregulating β_1_ and β_3_ integrins on the cell surface through an Akt, ERK, IKKα/β, NF-κB signalling pathway. The effects of bone morphogenetic protein-2 were blocked by kinase inhibitors or integrin knockdown [96], suggesting it to be a potential target in blocking the spread of bone metastasis.

α_v_β_3_ on osteoclasts also plays a significant role in the development of bone metastasis. Functional osteoclasts are required for both physiological and pathological bone remodelling, with pathological destruction of bone being necessary for a metastasis to gain space to develop. Knockdown of β_3_ in bone marrow, or other disruption of osteoclast function, protects mice from the development of osteolytic bone metastases, indicating that interaction between tumour cells and osteoclasts are required for tumour growth and bone destruction [31].

In a PC-3 model lacking α_v_β_3_, adhesion and migration on Vn was mediated by α_v_β_1_ and α_v_β_5_ [58]. siRNA knockdown of α_v_ caused regression of bone xenograft tumours, but had no effect on subcutaneous xenografts, indicating that integrin-mediated interactions with bone stroma are specifically required for the survival of bone metastasis. The ability of multiple RGD-binding integrins to support such interactions suggests that multi-integrin targeted drugs will be required for integrin-targeted anti-metastatic therapy to be effective. Interaction with the bone stroma has also been shown to promote resistance to radiotherapy, and treatment with the anti-α_v_ antibody CNTO-95 reversed this effect *in vitro* [97]. The mechanism of α_v_β_3_ induced radioresistance has been shown to involve maintaining high levels of survivin [98].

A large number of signalling pathways affect integrin expression thus inducing downstream effects in prostate cancer cells. For example, leptin signalling upregulates α_v_β_3_ expression and thus promotes cell migration. Increased cell migration in response to leptin can be blocked by inhibition of the kinase signalling pathway or by blocking integrin function [99]. High levels of calcitonin and its receptor correlate with tumour grade. Calcitonin activates the urokinase receptor (uPAR) and promotes migration on Vn through increased α_v_β_3_ levels and promotion of α_v_β_3_-uPAR association and FAK signalling [100]. Exposure of prostate cancer cells to prostaglandin E_2_ induces phosphorylation of EGFR, MEK, ERK1/2, and promotes expression and phosphorylation of β_3_ integrin [101]. Subsequent signalling promotes cancer progression through activator protein-1 transcriptional control, and cell migration and angiogenesis through increasing uPA and VEGF secretion. Chemokine signalling has been implicated in controlling prostate cancer cell invasion, α_v_β_3_ integrin clustering and localisation and thus cell adhesion [102].

α_v_β_6_ has not been extensively investigated in prostate cancer, but is implicated in progression of a number of other cancers including gynaecological, head and neck and colon adenocarcinoma, where it has been shown to promote invasion and metastasis through activation of MMPs (in a similar manner to α_v_β_3_) and activation of TGF-β1 [103]. α_v_β_6_ expression is associated with inflammation, and the β_6_ subunit is increased in cases of benign prostatic hypertrophy [39] as well as in prostate cancer, where α_v_β_6_ has been implicated in controlling prostate cancer growth in conjunction with the androgen receptor [56]. Increased transcriptional signalling from Stat3 in prostate cancer leads to increased expression of Fn and its receptor α_v_β_6_, thus increased cell migration, anchorage independent growth and epithelial mesenchymal transition (EMT), which were inhibited by an anti-α_v_β_6_ antibody [48]. Constitutive Stat3 activation is associated with tumour progression; integrin antagonism is a potential therapeutic target to reverse its effects. α_v_β_6_ has been shown to cooperate with α_v_β_1_ and α_5_β_1_ to allow cells to migrate on Fn. This allowed cells to maintain the ability to migrate when treated with an antagonist selective for 1 or 2 integrins [104].

Little is known regarding the role of α_v_β_1_ in cancer. α_v_β_1_ expression has been shown to be controlled by the amount of the α_v_ subunit available in the cell, and it is the first integrin to be downregulated as α_v_ levels decrease [105], therefore it may gain a more prominent role if other α_v_-containing heterodimers are suppressed.

α_v_β_8_ shares with α_v_β_6_ the ability to activate latent TGF-β. α_v_β_8_ has been shown to be a negative regulator of tumour growth in mouse models [106,107]. There is little data available on α_v_β_8_ expression in human tumours, and the relevance of the model studies to human disease remains to be determined.

A systems biology approach to prostate cancer has identified key proteins which act as links between multiple signalling networks essential for tumour progression. Hsp90, Runx2 and α_v_ integrin signalling have been identified as key to 3 networks, which are extensively interconnected to form a single supernetwork which will disintegrate when key molecules are targeted. α_v_ integrin signalling controls tumour growth at all stages of disease, and both α_v_ and Runx2 promote metastasis and tumour growth in the bone microenvironment [108]. This study provides strong support for a causal effect of α_v_ integrins in prostate cancer. The relative importance of individual members of the α_v_ subfamily in the signalling network remains to be identified, however, the use of multi-integrin antagonists targeting the whole subfamily should efficiently disrupt the prostate cancer signalling network thus prevent tumour growth and progression.

### 3.2. α_IIb_β_3_

The presence of α_IIb_β_3_ on the cell surface of DU145 cells is associated with increased tumourigenicity, local invasion, and development of lymph node metastases. In contrast, PC-3 cells with intracellularly-located α_IIb_β_3_ were less invasive. Antibodies to α_IIb_β_3_ prevented lung colonisation by DU145 cells in the mouse tail vein injection model of the early steps of metastasis [34].

α_IIb_β_3_ expression on platelets can mediate tumour-platelet interactions in the absence of tumoural α_IIb_β_3;_ tumour-platelet interactions have recently been reviewed by Buergy *et al.* [109]. Contact with tumour cells stimulated platelet activation and aggregation, forming tumour-platelet aggregates, which promote tumour cell arrest in the circulatory system, an intermediate stage of metastasis. Platelet aggregation induced by PC-3 cells has been prevented by the α_IIb_β_3_ antagonist peptide GRGDS and the disintegrins trigramin and rhodostomin [110]. Platelet activation provides a source of soluble growth factors for promoting tumour growth at the metastatic site, and promotes the release of platelet-derived microparticles. Exposure to platelet-derived microparticles caused cancer cells to become α_IIb_β_3_ expressing, increased angiogenesis and metastasis [111] and promoted invasion and MMP-2 production in prostate cancer cells [112].

α_IIb_β_3_ antagonism has the potential to prevent metastatic spread by multiple mechanisms; preventing tumour platelet interaction through action of tumour or platelet integrin receptors thereby reducing tumour cell survival in the bloodstream, or by preventing cell adhesion, invasion or growth at the metastatic site. The small molecule α_IIb_β_3_ antagonist ML464 has been shown to be effective at preventing bone metastasis and reducing visceral metastasis in a mouse model of melanoma, but has not yet been tested against prostate cancer [31].

### 3.3. α_5_β_1_

α_5_β_1_ has been shown to control the proliferation of prostate cancer cells in 3D culture through formation of a signalling complex with insulin-like growth factor type 1 receptor (IGF-1R) [113]. β_1_ integrin mediated binding to fibronectin protects DU145 cells against the cytotoxic effects of docetaxel, again through promoting IGF-1R-β_1_ complex formation [114]. However, fibronectin did not protect cells against radiotherapy, possibly because exposure to high dose radiation reduces β_1_ expression and associated cell adhesion [115].

α_5_β_1_ controls fibronectin matrix assembly which in turn controls the organisation and stability of the ECM. The ability of prostate cancer cells to assemble a fibronectin matrix is correlated with the ability to form cohesive aggregates and cellular α_5_β_1_ expression. High α_5_β_1_ levels and fibronectin matrix assembly are inversely correlated with invasiveness; loss of α_5_β_1_ allows cell detachment, leading to intravasation and metastasis [116]. Agonism of α_5_β_1_ has therefore been proposed as an anti-metastatic strategy and the MEK inhibitor AZD6244 has been shown to promote cell adhesion, possibly by activating α_5_β_1_ [116], however such an approach will require careful assessment for safety, since α_5_β_1_-mediated adhesion and angiogenesis could promote the establishment of metastases once cells have detached from the main tumour. Recent work has shown that the actin regulatory protein Mena is required for α_5_β_1_-mediated functions and signalling in fibroblasts [117]. Upregulation of Mena, and expression of a pro-invasive isoform of the protein have been associated with metastasis in a number of cancers [118], suggesting that further studies of Mena may prove significant in the diagnosis and treatment of metastasis in future.

Tumour cell α_5_β_1_ and α_v_β_3_ mediate adhesion of prostate cancer cells to endothelial cells through binding cell surface fibronectin and vitronectin [67]. α_5_β_1_ and α_v_β_3_ have contrasting roles in the early stages of adhesion; α_5_β_1_ mediated cell adhesion to and spreading on fibronectin, and whereas inhibition of α_v_β_3_ had no effect on adhesion to and spreading on fibronectin, but promoted cell shape changes and reorganisation of cytoskeletal proteins, suggesting that α_v_β_3_ is a negative regulator of other fibronectin-binding integrins [65]. RGD peptides such as cRGDfV [67] and GRGDSP [65] were more effective at preventing cell attachment and spreading than specific anti-integrin antibodies, again demonstrating the limitation of selective integrin antagonists versus pan-integrin antagonists as anticancer agents. RGD peptides were also effective at preventing adhesion of prostate cancer cells to bone marrow endothelial cells, which express high levels of α_5_β_1_ [119].

Expression of α_5_β_1_ on prostate cancer cells is affected by a number of signalling pathways linked to tumour invasion and bone metastasis: the increase in cell adhesion to the endothelium caused by CXCL12 binding CXCR4 is a result of increased expression of α_5_ and β_3_ [71]. Parathyroid hormone related protein increased cell adhesion and promoted cell survival by upregulating cell surface levels of α_5_, along with α_1_, α_6_ and β_4_ subunits [120,121]. EGF also increased cell adhesion to fibronectin and increased migration by increasing cell surface levels of the β_1_ subunit without a corresponding increase in α_5_ [122].

Re-expression of the androgen receptor in androgen resistant DU145 cells increased α_5_ levels on the cell surface and cells’ invasive ability, but reduced cell adhesion to a number of ECM substrates including fibronectin [123]. Expression of Snail, a transcription factor associated with the epithelial mesenchymal transition, resulted in reduced cell adhesion and increased migration with decreased cell surface expression of α_5_ and β_1_ [124]. Snail has also been shown to increase α_v_β_3_ expression, and promote adhesion and migration to α_v_β_3_ ligands such as OPN, suggesting it controls in site-specific relocation of cancer cells [125].

Alternative splicing of the β_1_ integrin subunit gives rise to variants with different properties in cancer cell lines; the normal β_1_ (β_1A_) subunit is found in integrins which promote prostate cancer cell proliferation, whereas the β_1C_ variant inhibits cell proliferation. The expression of both β_1_ integrin variants is decreased at the mRNA level in prostate carcinoma, but only β_1C_ protein expression is lost due to reduced transcription and translation, and increased degradation of β_1C_, combined with increased translation of β_1A_ [126,127]. Loss of β_1_ integrin expression in androgen sensitive prostate cancer is reversible with short term neoadjuvant androgen deprivation therapy: expression of β_1C_ mRNA was significantly increased in patients treated with androgen deprivation therapy due to increased transcription [128]. The extent of reversal was negatively correlated with the tumours’ Gleason grade. No effect was observed with longer-term treatment [127], suggesting that β_1C_-mediated pathways are linked to androgen-mediated mechanisms and the development of resistance.

### 3.4. Angiogenesis

α_v_ integrins play an important role in developmental angiogenesis, with α_v_ knockdown being lethal in mice. This is possibly due to the effect of removing α_v_β_8_ integrin expression on brain development [129], since mice lacking β_3_ or β_5_ develop normally and subsequently show enhanced pathological angiogenesis [130].

Elevated expression of α_v_β_3_ and α_v_β_5_ on active endothelial cells, and the efficacy of their antagonists in preventing angiogenesis in preclinical studies [131,132] has led to these integrins becoming popular targets for anti-angiogenic drug development. However, subsequent work has revealed the complexity of integrin function in this area, with multiple integrins on multiple cell types interacting to both promote and prevent angiogenesis [27,133].

α_5_β_1_ has a similar role to α_v_ integrins during angiogenesis, with global α_5_ knockout being lethal; in endothelial cells, knockout of α_5_ alone had little effect on developmental angiogenesis due to compensation by α_v_, but knockout of both α_5_ and α_v_ integrins was lethal due to defects in vascular remodelling [134]. The use of non-selective integrin antagonists such as echistatin, or combination of selective α_5_β_1_, α_v_β_3_ and α_v_β_5_ inhibitory antibodies completely blocked tube formation by endothelial cells in a fibrinous exudate mimicking tumour stroma, whereas a selective α_5_β_1_ or α_v_β_3_ antibody had little effect [135]. In contrast, selective α_5_β_1_ antagonism has been shown to prevent lymphangiogenesis whilst allowing α_v_ integrin mediated angiogenesis [136]. These results suggest that multi-integrin antagonism will be required for efficient anti-angiogenic therapy in tumours, and suggest a possible explanation for the limited efficacy so far observed in clinical trials of α_v_β_3_/α_v_β_5_-targeted antiangiogenic agents.

### 3.5. Epithelial-Mesenchymal Transition

TGF-β upregulates expression of the RGD-binding integrins [137] and there is extensive crosstalk between TGF-β and α_v_β_3_ [138], α_v_β_5_ [139,140], α_v_β_6_ [103,141], and α_5_β_1_ [142,143]. TGF-β also regulates EMT, where cells acquire the invasive, migratory phenotype required for metastasis. EMT is associated with increased α_v_ expression [139,144]. In cancer, α_v_β_3_ [145], α_v_β_5_ [139] and α_5_β_1_ [146] have been shown to be important in allowing EMT to take place, and α_v_β_6_ is induced during the process [147]. Inhibition of RGD-binding integrins has been shown to cause invasive prostate cancer cells to revert to a non invasive epithelial phenotype [148], and to block EMT in other cancers [139].

The importance of TGF-β signalling [149] and EMT [144] in bone metastasis and the role of the upregulated integrins in promoting tumour invasion and metastasis [137] suggests the associated signalling pathways as a therapeutic target in advanced prostate cancer. However, most research linking EMT and integrin function is in non-prostate cancers, and specific roles in prostate cancer, and particularly in the mesenchymal to epithelial reverse transformation observed in established metastasis [97], remain to be established.

### 3.6. Laminin Binding Integrins

Changes in expression of other integrins containing the β_1_ subunit is also characteristic of prostate cancer progression. Expression of α_2_β_1_ [150] and the laminin binding integrin α_3_β_1_ [42] have been associated with disease progression and metastasis. Increased α_6_ surface expression [151,152] and a switch in the heterodimeric partner of α_6_ from α_6_β_4_ to α_6_β_1_ [152] promotes cell migration and invasion. The α_6_ subunit is also cleaved by urokinase plasminogen activator, [153,154,155] to give the cancer-specific shortened subunit α_6_p which promotes metastasis. Prevention of α_6_ cleavage reduces prostate cancer cell migration and invasion, and reduces the development and effects of bone metastasis [156,157].

β_1_-containing integrins can interact with other RGD-binding integrins via trans-dominant inhibition, where expression of one integrin suppresses function of another by competing for intracellular activator proteins, or changes the expression of other integrins by altering mRNA stability and translation [158]. Some examples of integrins interacting *via* transdominant inhibition include function modulating crosstalk between α_v_β_5_ and α_5_β_1_ [159], α_v_β_3_ or α_v_β_5_/α_v_β_6_ and α_2_β_1_ [160,161], and α_v_β_3_ and α_3_β_1_ or α_6_β_1_ [162]. Introducing β_1_ expression in a model system has been shown to decrease β_3_ and increase β_5_ protein levels [158]. The α_v_β_3_ antagonist cilengitide has been shown to reduce β_1_-mediated endothelial cell adhesion and survival, since it is able to activate α_v_β_3_ as well as block the α_v_β_3_ adhesion site [163]. The significance of such interactions has not yet been explored in prostate cancer, and there is a need for more basic research in the general area of trans-dominant inhibition. From a translational perspective, the current data further emphasises the importance of fully characterising integrin expression in model systems so the presence of trans-dominant inhibition can be included in interpretations of results.

## 4. Changes in Integrin Expression in Response to Therapy

The interconnected nature of cell signalling pathways means that drugs targeting other receptors and kinases can affect the expression and function of integrins through modulating their transcription, transport or downstream signalling. Studies in this area are summarised in Table 5.

**Table 5:** Alterations in cell surface expression of integrin subunits in response to treatment with non-integrin targeting drugs.

Drug	Cell line	Integrin subunits upregulated	Integrin subunits downregulated	Reference
Zoledronic acid	PC-3	β_1_	β_4_	[164]
Doxazosin	PC-3	β_8_ (mRNA)	α_5_, α_v_, β_1_, β_5_, β_6_ (mRNA)	[165]
Camptothecin	PC-3		α_v_, β_3_, β_5_	[166]
Genistein	DU145	α_2_, α_3_, β_1_		[167]
Valproic acid (VPA)	PC-3	α_1_, α_3_	α_6_, β_3_	[168,169,170,171]
VPA + interferon-α	PC-3	α_1_, α_3_, β_1_	α_5_, α_6_, β_4_	[168]
AEE788	PC-3	α_1_	α_5_	[70,170,171]
RAD001	PC-3	α_2_, β_3_	α_1_, α_5_	[70,169,171]
AEE877+ RAD001	PC-3	α_2_, β_3_	α_1_, α_5_	[70]
VPA+RAD001	PC-3	α_1_, α_2_, α_3_	α_5_, α_6_, β_3_	[169]
VPA+AEE788	PC-3	α_1_, α_3_	α_5_, α_6_, β_3_	[170]
VPA+AEE788+RAD001	PC-3	α_1_, α_2_, α_3_, β_1_	α_5_, α_6_, β_3_	[171]
VPA	LNCaP	α_2_, α_3_, α_5_, α_6_, β_1_		[168,169,170,171]
VPA + interferon-α	LNCaP	α_2_, α_3_, α_5_, α_6_, β_1_, β_3_, β_4_		[168]
AEE788	LNCaP	α_3_	α_2_, α_6_	[70,171]
RAD001	LNCaP	α_3_	β_1_	[70,169,171]
AEE877+ RAD001	LNCaP	α_3_, α_5_, α_6_, β_1_		[70]
VPA+RAD001	LNCaP	α_2_, α_3_, α_5_, α_6_, β_1_		[169]
VPA+AEE788	LNCaP	α_2_, α_3_, α_5_, α_6_, β_1_		[170]
VPA+AEE788+RAD001	LNCaP	α_2_, α_3_, α_5_, α_6_, β_1_		[171]

A number of molecularly targeted drugs with different mechanisms of action have been assessed for their effects on integrin expression as well as on prostate cancer cell growth and motility. The effects of RAD001 (mTOR inhibitor), VPA (HDAC inhibitor), and AEE877 (EGFR/VEGFR inhibitor) have been extensively investigated by Wedel *et al.* [70,168,169,170,171]. These drugs inhibited adhesion of all cell lines tested, and inhibited migration and invasion of β_3_-expressing PC-3 cells but had lesser effect on β_3_ negative LNCaP cells [70,169]. Changes in integrin expression and localisation in response to treatment were cell line dependent; β_3_ and β_4_ were often downregulated in cell lines that originally expressed them, and β_4_ was relocated from the cell surface to an intracellular distribution in PC-3 cells [168,169,170,171]. Initial expression of the β_3_ and β_4_ subunits was proposed as a biomarker predicting response to treatment. Changes in expression of β_3_ and β_1_ and associated α subunits including α_5_ may promote anti-invasive and anti-metastatic effects. RAD001 alone was a less effective anti-adhesive agent than the other compounds tested and was found to upregulate β_3_, suggesting potential issues with RAD001 promoting drug resistance and metastasis [70].

Camptothecin, and the anti-angiogenic camptothecin-somatostatin conjugate JF-10-81, reduced expression of functional α_v_β_3_ and α_v_β_5_ on PC-3 cells, as well as reducing PI3K/Akt signalling and expression of MMP-2 and MMP-9 [166]. These results suggest that the anti-migration and anti-invasive effects of camptothecin may result from interference with integrin-mediated signalling. Further work is required to determine the mechanism by which camptothecin downregulates α_v_β_3_/α_v_β_5_ expression.

Exposure of LNCaP cells to androgen stimulation and radiation resulted in increased cell surface expression of the α_v_ and β_1_ integrin subunits resulting in increased adhesion to fibronectin, which may provide a mechanism for radioresistance [172]. In patients with locally advanced disease, the anti-androgen bicalutamide adjuvant to radiotherapy demonstrates significant clinical benefits in terms of overall survival, PFS and PSA-PFS compared with radiotherapy alone [173]. Since bicalutamide normalized integrin expression, this could provide a mechanism by which this agent increases tumour radiosensitivity. Furthermore, use of an integrin antagonist could provide beneficial anti-adhesion effects without the side-effects and risk of promoting androgen independent disease seen with hormone therapy.

Addition of physiological levels of fish oil, lycopene, or vitamin E to cell culture medium decreased cell surface expression of α_v_β_3_ and α_v_β_5_ on PC-3 cells [174]. The role of vitamin E in prostate cancer is complex since it has been shown to promote tumour growth, however lycopene and fish oil have been linked with decreased cancer risk, and may have potential as adjuvant nutrients.

Treatment of PC-3 cells with the α_1_-adrenoceptor antagonist doxazosin caused a significant decrease in the levels of α_5_, α_v_, β_1_, β_5_ and β_6_ mRNA, accompanied by an increase in β_8_ [165]. Doxazosin induced apoptosis, possibly by interfering with cell attachment and integrin mediated survival signalling.

Changes in integrin expression in response to chemotherapy can be exploited to increase response to anti-integrin therapeutics [175]. However, changes in expression could also render a targeted therapeutic ineffective therefore incorporation of an integrin antagonist into existing or novel drug combinations will require prior assessment of their effects on integrin expression at the preclinical stage. Measurement of changes in integrin expression may also prove useful as biomarkers, for example, in detecting upregulation of pro-tumourigenic integrins as an indicator of the limit of clinical effectiveness of a particular therapy, or the development of resistance.

## 5. RGD-Binding Integrin Antagonists in Prostate Cancer Therapy

### 5.1. Preclinical Studies

Overexpression of integrins α_v_β_3_, α_v_β_5_ and α_5_β_1_ on tumour tissue and angiogenic vasculature, and the associated consequences on tumour progression and/or resistance, makes them attractive targets for molecularly targeted therapy. In addition, involvement of osteoclast α_v_β_3_ in bone remodelling provides further incentive for developing α_v_β_3_ antagonists as novel therapies for osteoporosis and bone metastasis. In recognition of the importance of the integrins in promoting cancer progression, integrin antagonist antibodies, peptides and small molecules have been developed for a wide range of anti-tumour applications. Here, we review antagonists of RGD-binding integrins specifically relevant to prostate cancer.

The importance of targeting integrins in the tumour microenvironment including on endothelial cells in addition to those on tumour cells was demonstrated using α_v_β_3_/α_IIb_β_3_ negative PC-3 cells grown in an α_v_β_3_-positive bone microenvironment [176]. Antagonism of β_3_ integrins with m7E3 F(ab')_2_ reduced tumour growth, angiogenesis, and tumour-induced bone degradation. This demonstrated that targeting RGD-binding integrins, specifically the β_3_ subfamily will have therapeutic benefit against bone metastasis regardless of tumour integrin profile, particularly important in light of recent demonstrations of heterogeneity of genes and receptors expressed by tumour cells within single patients [177].

Integrin knockdown has confirmed the importance of targeting integrin-mediated interactions with the microenvironment and that the α_v_ integrin subfamily is a target for the prevention and treatment of bone metastasis. In studies progressing the development of GLPG0187, knockdown of α_v_ in PC-3 cells reduced clonogenicity and prevented the growth of subcutaneous tumour xenografts *in vivo* [178]. A gene therapy approach using liposomes to deliver α_v_-siRNA to established tumours showed that α_v_-knockdown had no effect on the growth of established subcutaneous PC-3 xenografts, but significantly decreased the growth of PC-3 xenografts in bone and associated bone destruction, indicating α_v_ integrin mediated communication with the bone microenvironment is required for the development of bone metastases [58].

The small molecule multi-integrin antagonist GLPG0187 has been reported to have a low nanomolar affinity for RGD-recognising integrins, with the exception of α_IIb_β_3_ [148]. GLPG0187 is a potent anti-angiogenic and anti-osteoporotic agent, and inhibits *in vitro* adhesion and migration of prostate cancer cell lines. GLPG0187 treatment caused an increase in the E-cadherin/vimentin ratio, indicating a switch to an epithelial phenotype, and caused a decrease in the subpopulation of prostate cancer stem cells. *In vivo*, dosing with GLPG0187 reduced the tumour burden developed in mice injected intracardiacally with PC-3M-Pro4/luc cells as a model of the development of new bone metastases. Treatment of mice with established bone metastases significantly reduced the growth of existing tumours and prevented the formation of new metastases [148,178]. These studies identify GLPG0187 as a promising treatment for metastatic prostate cancer, and it has been shown to be safe in healthy volunteers. A Phase 1b trial in advanced solid tumours is currently in progress [179].

The small molecule integrin antagonist S247 has been described as an α_v_β_3_ antagonist, but actually has low nanomolar affinity for all α_v_ integrins [180]. S247 has been effective at preventing bone metastasis in mouse models of breast cancer [180,181], and has been investigated as a combination therapy with radiation, to combat the increase in pro-survival integrin signalling occurring when α_v_β_3_ is upregulated in response to radiation [182]. A PC-3/HUVEC co-culture model was used to model tumour-endothelium interactions; S247 and radiation showed an enhanced reduction in cell proliferation and survival compared to single therapies, and prevented HUVEC migration induced by irradiation of PC-3 cells. In a PC-3 xenograft model, monotherapy with S247 significantly reduced tumour growth, and combination with radiotherapy resulted in a greater delay in tumour growth and, in some cases, caused tumour regression [182].

The Ac-PHSCN-NH_2_ peptide, ATN-161, was developed as an antagonist of the PHSRN synergy sequence of fibronectin which acts as a promoter of DU145 cancer cell invasion through binding to α_5_β_1_ [183]. PHSCN covalently binds the β subunits of α_5_β_1_, α_v_β_3_ and α_v_β_5_ by forming disulfide crosslinks with the integrin [184] and prevents cancer cell invasion independent of effects on adhesion. ATN-161 was a nanomolar inhibitor of α_5_β_1_-expressing MLL prostate cancer cell invasion *in vitro*, and effectively suppressed tumour growth, angiogenesis and metastasis *in vivo* [183]. Notably, ATN-161 suppressed the development of metastases when treatment was started after resection of primary MLL tumours [183], a model highly relevant to adjuvant treatment in the clinic. Treatment started prior to the removal of DU145 tumours completely prevented recurrence of the primary tumour and significantly reduced metastasis [185]. A polylysine dendrimer, Ac-PHSCNGGK-MAP, bearing 8 PHSCN peptides has been shown to be significantly more potent than ATN-161, with synergistic enhancements of inhibition of fibronectin-induced invasion of PC-3 or DU145 cells *in vitro*, and inhibition of cell extravasation and development of micrometastases in the lungs *in vivo* [186] To date, studies on PHSCN peptides have focussed on combating lung metastasis. Investigation of PHSCN peptides effects on bone metastasis will be important in determining whether they will prove to be an effective general treatment for advanced disease. ATN-161 has been progressed to phase 1 clinical trials including 4 patients with prostate carcinoma; it was well-tolerated and one patient with prostate cancer experienced prolonged stabilisation of disease [187].

Disintegrins are a family of small peptide integrin antagonists derived from snake venom [188]. Many contain RGD or related motifs and act as antagonists of the RGD-recognising integrins, blocking platelet aggregation and integrin mediated cell adhesion. Other disintegrins contain different integrin binding motifs such as LDV or MLD, permitting them to antagonise other β_1_ family integrins. The high anti-integrin activity of disintegrins has led to interest in their development as potential anti-thrombotic or anti-metastatic agents. Disintegrins including rhodostomin, triflavin and trigramin have been shown to inhibit α_IIb_β_3_-mediated tumour cell induced platelet aggregation [110], α_v_β_3_-mediated adhesion of PC-3 cells to the extracellular matrix produced by osteoblasts [189], and tumour growth in bone [189], suggesting they may be useful as a treatment for bone metastasis.

The disintegrin contortrostatin has been shown to bind a number of RGD-containing integrins, including α_v_β_3_, α_IIb_β_3_, α_v_β_5_ and α_5_β_1_ (Binding K_d_: α_v_β_3_ 6.6 nM, α_v_β_5_ 19.5 nM, α_5_β_1_ 191.3 nM [190]), rendering it able to prevent invasion and tumour growth of cancer cells expressing a range of integrin profiles [191]. Contortrostatin inhibited PC-3 cell migration and enhanced the cytotoxicity of docetaxel *in vitro* [59]. In subcutaneous xenograft models, contortrostatin alone significantly decreased growth of both androgen dependent (CWR-22) and independent (PC-3) tumours, and an increased effect was obtained in combination with docetaxel. Contortrostatin and combination therapy was also effective at preventing the growth of orthotopic bone lesions from PC-3. Further investigations are required to determine whether the enhanced efficacy of docetaxel with contortrostatin is simply additive or whether there is a synergistic effect. Nevertheless, the observation that disintegrins can both enhance the effect of a currently used chemotherapy drug and affect the development of bone metastasis, strongly encourages further research on these proteins. The availability of recombinant disintegrins [190,192] provides a substantial translational potential for their use as biopharmaceuticals; they are now readily produced in large quantities, and mutated variants can be produced to explore integrin affinity and antitumour efficacy.

### 5.2. Clinical Trials

The cyclic peptide cRGDfV has been shown to be cytotoxic to α_v_β_3_-expressing prostate cancer cells, inducing apoptosis by interference with integrin-mediated FAK signalling [63]. No effects were observed on cells which did not express α_v_β_3_, therefore cRGDfV was proposed as a non-toxic targeted therapy for prostate cancer. The most advanced integrin antagonist in clinical development, cilengitide, is a *N*-methylated cRGDfV derivative, c(RGDf(NMe)V) with high affinity for α_v_β_3_, α_v_β_5_ and α_5_β_1_ [193,194,195]. *In vitro*, cilengitide has been shown to be highly effective at blocking cell adhesion and migration mediated by α_v_β_3_ and α_v_β_5_ [194], and induced apoptosis in cancer cells dependent on integrin mediated cell adhesion for survival [196]. *In vivo*, cilengitide reduced angiogenesis, tumour growth and tumour metastasis [197]. A preclinical study has shown that low doses of cilengitide have a paradoxical effect promoting tumour growth [198]. Although the relevance of this observation to conditions in the clinic has been questioned [199], it has been suggested that the dose level or timing of treatment will require careful optimisation.

At least 21 clinical trials involving cilengitide have been completed or are ongoing, the most notable being a current Phase III trial for the treatment of glioblastoma. Phase I studies on advanced solid tumours [200,201] showed cilengitide was safe and well-tolerated. No patients experienced partial or complete response to treatment, although some achieved prolonged stable disease. Subsequently, cilengitide has been tested in two Phase II trials against metastatic and non-metastatic prostate cancer [202]. 13 patients with non-metastatic castration resistant prostate cancer were treated with 2000 mg cilengitide administered intravenously twice weekly [203]. Cilengitide was well tolerated but no patients met the primary endpoint of a decrease in PSA level of ≥50%, indeed 11 experienced an increase in PSA level over the course of the trial, resulting in the conclusion that cilengitide had no detectable activity. 44 patients with asymptomatic metastatic castration resistant prostate cancer were treated with either 500 or 2,000 mg cilengitide administered intravenously twice weekly [204]. Some clinical effect was observed, with 7 patients experiencing stable disease (average duration 9.9 months). All patients had an increase in biomarkers of bone turnover, but this was less pronounced in those receiving the higher dose, and those with stable disease.

MK-0429, a small molecule α_v_β_3_ antagonist, has been investigated in a phase I trial of patients with castration resistant prostate cancer and bone metastases [205]. 21 patients received either 200 mg or 1,600 mg MK-0429 twice daily for 4 weeks. This short duration of treatment made it difficult to draw conclusions about drug efficacy. The drug was safe, well-tolerated, and showed effects on bone turnover with a significant reduction in uNTx, a biomarker of bone resorption. However, all patients had an increase in PSA.

DI17E6, a monoclonal antibody targeting α_v_ integrins [206] has been investigated as a salvage therapy for patients whose disease progressed on standard chemotherapy. In a phase I trial of patients with castration resistant prostate cancer and bone metastases, 26 patients were treated with DI17E6 every 2 weeks for between 14 and 534 days. Two patients had a significant response with a marked decrease in PSA levels, and pain relief. One of these showed a reduction in size of the primary tumour and lymph nodes [207,208].

Another anti-α_v_ monoclonal antibody, intetumumab (CNTO 95) has been investigated as a combination therapy for metastatic castration resistant prostate cancer. 10 patients were treated with a combination of 5 or 10 mg/kg intetumumab combined with 75 mg/m² docetaxel. 5 mg/kg intetumumab was not effective, whereas no progressive disease occurred in the 10 mg/kg group, with 1 patient having partial tumour response and 4 having a decrease in PSA levels [209]. The anti-α_v_β_3_ antibody etaracizumab (Abegrin, Medi-522) has been investigated in a phase II clinical trial as a treatment for metastatic castration resistant prostate cancer in combination with docetaxel, prednisone, and zoledronic acid [179]. Results from this study have not been reported.

Conclusions on the clinical efficacy of integrin antagonists in prostate cancer are limited due to the small size of all studies conducted so far. Integrin antagonists are generally well-tolerated, and antibodies targeting a range of α_v_ integrins have shown some efficacy against advanced prostate cancer.

## 6. Integrin Targeting for Drug Delivery and Imaging

The overexpression of integrins, specifically α_v_β_3_, on a wide range of tumour cells and vasculature has made α_v_β_3_ a popular target for the development of agents for tumour detection and drug delivery. The most common strategy is use of a cyclic RGD containing peptide with high affinity to α_v_β_3_, and possibly also α_v_β_5_. The majority of RGD-targeted imaging agents are first developed in models of glioma, since U87MG expresses very high levels of α_v_β_3_ and α_v_β_5_. The wider field of integrin imaging and targeting in cancer has been recently reviewed in *Theranostics* [210,211]. Here, we discuss approaches specifically targeting prostate cancer.

### 6.1. Drug Delivery

cRGDfK peptide has been used to direct HPMA-copolymer geldanamycin conjugates to prostate cancer cells and tumour vasculature to avoid the dose-limiting toxicity associated with current therapeutic use of geldanamycin [212]. α_v_β_3_-expressing PC-3, DU145 and HUVEC cells were used for initial assessment of the conjugates’ anticancer activity, showing activity comparable to or greater than the free drug whereas a geldanamycin-free cRGDfK-targeted polymer showed cytotoxic effects at high concentrations. α_v_β_3_-targeted copolymers significantly inhibited HUVEC migration and tube formation [213] whereas cRGDfK conjugate without geldanamycin or cRGDfK alone had no effect on HUVEC migration. *In vivo*, α_v_β_3_ targeting of geldanamycin doubled the tolerable dose in nude mice. The conjugate effectively delivered drug to DU145 xenografts, providing an intratumoural drug concentration over 8 times higher than that provided by the equivalently tolerated dose of free geldanamycin [214]. Xenograft growth and *in vivo* angiogenesis were effectively inhibited. Subtherapeutic doses of α_v_β_3_-targeted conjugate increased tumour tissue permeability, which could be exploited to improve drug delivery of a second agent by use of the cRGDfK targeting agent in a combination therapy [213].

cRGDfK has also been used as a targeting group to deliver other payloads. HPMA-copolymer docetaxel conjugates have been investigated in models of prostate cancer in order to address toxicity and solubility issues seen with current docetaxel formulations [215]. The copolymers are water soluble and combine active tumour targeting with improved renal elimination of conjugated drug in the systemic circulation. *In vivo*, α_v_β_3_-targeted copolymer caused no acute toxicity and caused a significantly greater reduction in DU145 xenograft tumour growth compared to docetaxel alone. Nanoparticles containing Pt(IV) prodrugs targeted by cRGDfV are more cytotoxic than cisplatin in PC-3 and DU145 cell lines [216], but have not yet been tested in *in vivo* models of prostate cancer, although they proved to be effective in suppressing tumour growth in a breast cancer model. cRGDfV-functionalised gold nanorods bound selectively to α_v_β_3_-expressing cells *in vitro*, but failed to localise to DU145 tumours *in vivo* due to rapid clearance [217].

The RGD4C α_v_β_3_-binding peptide has been used to deliver HPMA-copolymers chelating β-emitting isotopes for tumour-targeted radiotherapy [218]. Imaging of tumour angiogenesis was also possible via chelation of ^99m^Tc and showed specific and enhanced uptake in DU145 and PC3 xenografts [218,219], and prolonged retention of the conjugated isotope [218]. Chelation of ^90^Y resulted in significant inhibition of DU145 tumour growth with no evidence of radiation injury to non-target organs [218].

Linear CRGDC has been conjugated to the *N*-terminus of tachyplesin, a 17 amino acid antimicrobial cyclic peptide produced by the horseshoe crab which triggers apoptosis in cancer cells. RGD-tachyplesin reduced the proliferation and colony formation of TSU prostate cancer cells *in vitro*, increased cell permeability by causing damage to the plasma membrane, and initiated apoptosis through both Fas-dependent and independent pathways. *In vivo*, RGD-tachyplesin inhibited TSU xenograft growth in the CAM model without toxicity to the embryo [220].

The iRGD peptide has been used to promote tumour penetration of imaging agents and drugs. iRGD contains a cyclic RGD motif which first binds to tumour cells via α_v_ integrins then undergoes proteolytic cleavage to reveal a linear CendR fragment with affinity for neuropilin-1 which allows cell and tissue penetration. iRGD has high affinity for α_v_β_3_ and α_v_β_5_ but not α_5_β_1_ and is localised to regions overexpressing both α_v_ and neuropilin-1 in 22Rv1 orthotopic prostate cancer xenografts. iRGD-coating of abraxane increased abraxane accumulation in 22Rv1 tumours 8-fold compared to the untargeted drug, whereas CRGDC-targeting only afforded a 2-fold increase, and effectively inhibited tumour growth. Additionally, conjugation to iron oxide nanoworms allowed MRI imaging of the whole tumour region, a significant improvement compared to the linear CRGDC peptide which only targeted tumour vasculature, or untargeted nanoworms which gave no signal in the tumour [221]. Use of iRGD for drug delivery provides a substantial advantage over other integrin-targeting peptides due to its greater tissue penetration promoting capabilities.

A fusion protein containing a linear RGD peptide attached to the Fc fragment of mouse IgG has been used in gene therapy to target α_v_β_3_ expressing tumours, and tumour angiogenesis through α_v_β_3_ expressing endothelial cells [222]. The selectivity of the peptide used for α_v_β_3_ over other RGD-binding integrins was not reported. *In vivo*, injection of an adenovirus vector causing expression of the fusion protein significantly decreased the growth of DU-145 xenografts, and similar growth suppression was observed in tumours distant from the treated site. The fusion protein can be used as a targeted therapeutic separate from its gene therapy applications, thus may have potential as a treatment of metastatic prostate cancer.

A fibronectin mimetic peptide containing RGDSP and PHSRN sequences has been developed to deliver liposomes [223] or polymer vesicles [224] to α_5_β_1_-expressing prostate cancer cells. Including the PHSRN sequence directed the peptide specifically to α_5_β_1_ and increased efficiency of internalisation and drug delivery to LNCaP cells compared to GRGDSP. α_5_β_1_-targeting of polymer vesicles containing TNF-α increased cytotoxicity 2.6-3.8-fold compared to free TNF-α [224]. *In vivo* applications of this strategy have not yet been reported.

### 6.2. Imaging

The low metabolic activity observed in prostate cancer has led to ^18^F-fluorodeoxyglucose-based PET scanning being found unsuitable for diagnosis and staging [225]. Targeting of positron emitting agents to integrin receptors overexpressed on the tumour has been proposed as one method of overcoming this problem [226]. Other PET imaging approaches for the detection and monitoring of primary and metastatic prostate cancer have been reviewed by Cai *et al.* in 2010 [227].

Initial investigations of α_v_β_3_-targeted PET imaging in prostate cancer models raised some concerns that the level of α_v_β_3_ on tumour tissue was insufficient to allow good imaging, for example, in a study of an ^18^F-labelled peptide bearing two cRGDyK targeting groups, PC-3 tumours had a low to moderate level of α_v_β_3_ expression resulting in low tumour uptake [228]. In preliminary clinical studies, ^18^F-galacto-RGD detected 78% of bone metastases, but uptake was heterogeneous, indicating that α_v_β_3_ levels differ between patients and metastatic sites [229]. 

Integrin targeting has been used in dual targeting approaches to enhance tumour binding and uptake. A range of molecules targeting both α_v_β_3_ and the gastrin releasing peptide receptor (GRPR), which is highly expressed on androgen resistant prostate tumours, have been investigated. ^18^F-FB-BBN-RGD contains cRGDyK and a bombesin (BBN) peptide analogue as targeting groups [230] and its uptake in PC-3 cells and tumour depended partially on both α_v_β_3_ and GRPR receptors. In xenograft models, a synergistic enhancement of imaging properties was observed: tumour uptake was increased compared to ^18^F-FB-RGD in PC-3 tumours, and washout from the tumour was decreased as a result of dual receptor binding. ^18^F-FB-BBN-RGD was also effective at imaging DU145 tumours with low GRPR levels, and showed reduced background signal compared to ^18^F-FB-RGD. ^18^F-FB-BBN-RGD comprised a mixture of two inseparable regioisomeric compounds, and was therefore thought unsuitable for clinical development [231].

The structure of RGD-BBN dually targeted imaging agents has been modified to improve efficiency of the chemical synthesis. ^18^F-FB-PEG_3_-Glu-RGD-BBN incorporated a PEG linker between the peptide and ^18^F label to improve radiolabelling efficiency and hydrophilicity, and was obtained as a single isomer. ^18^F-FB-PEG_3_-Glu-RGD-BBN was more effective than a similar molecule bearing 2 cRGDyK motifs at imaging PC-3 tumour xenografts [232]. A symmetrical linker (AEADP) has also been developed to afford a straightforward chemical synthesis yielding a single compound with defined structure [231]. ^18^F-FB-AEADP-BBN-RGD showed high and rapid uptake, and good tumour-to-normal tissue contrast in PC-3 tumour xenograft-bearing mice. The AEADP linker provides a platform technology to allow the rapid synthesis of agents bearing different receptor-targeting peptides.

Imaging with ^64^Cu has been investigated using NOTA and DOTA-bearing cRGDyK-BBN tracers [233]. The NOTA-functionalised peptide had better radiochemical labelling, PC-3 tumour uptake and tumour-to-normal tissue ratios than the DOTA-containing variant. Tumour uptake was slower than ^18^F-FB-PEG_3_-Glu-RGD-BBN, but ^64^Cu-NOTA-RGD-BBN allowed imaging over a longer timeframe due to the longer half-life of ^64^Cu vs ^18^F. The high selectivity for tumour tissue and long tumour retention time of ^64^Cu-NOTA-RGD-BBN suggests this agent may be useful for targeting radiotherapy to prostate tumours.

^64^Cu-NODA-RGD-Glu-6-Ahx-BBN(7-14)NH_2_ has been developed to overcome the problem of long retention times in the kidney observed with charged Cu-conjugates such as NOTA [234]. ^64^Cu-NODA-RGD-Glu-6-Ahx-BBN(7-14)NH_2_ showed high and diagnostically useful tumour uptake and tumour-to-normal tissue ratio allowing high quality imaging of PC-3 tumours over a long time period. The agent showed low affinity for α_v_β_3_ *in vitro*, suggesting initial tumour binding occurs through GRPR, and binding of the RGD peptide facilitates subsequent tumour accumulation and retention and urinary excretion.

Combined targeting of α_v_β_3_, nucleolin and tenascin-C has been used as an alternative strategy to enhance tumour imaging [235]. The Simultaneously Multiple Aptamers and RGD Targeting (SMART) probe used a RGD peptide to add α_v_β_3_ targeting capability to magnetic nanoparticles functionalised with fluorescent and Gd-chelating groups for multimodal imaging. The SMART molecule had significantly increased binding efficiency to DU145 cells compared to singly targeted nanoparticles, though it is interesting that a RGD-targeted nanoparticle was the most efficient singly targeted agent in this system.

cRGDfK has been used to target PGA-cystamine-Gd-DOTA conjugates for MRI imaging of α_v_β_3_ expression [236]. Quantitative T_1_ measurement detected high binding of the targeted agent at the periphery of DU145 tumour xenografts.

Cyanoacrylate microbubbles functionalised with RGD peptide have been used in ultrasound image of the response of AT-1 hormone independent Dunning rat prostate tumours to irradiation [237]. α_v_β_3_-targeted microbubbles showed enhanced accumulation in tumours compared to untargeted or nonsense peptide coated microbubbles. Following ^12^C irradiation, a significant increase in retention of α_v_β_3_-targeted microbubbles was observed, confirming previous reports that α_v_β_3_ is upregulated in response to radiotherapy. This approach has potential for monitoring tumour response to therapy and developing individualised scheduling of drug or radiation delivery. A related use of targeted microbubbles for the detection of prostate cancer has entered clinical trials [238].

Pseudopeptide small molecules containing the RGD tripeptide sequence cyclised with an azabicycloalkane unit have been conjugated with fluorescein isothiocyanate to afford non-cytotoxic tracers for α_v_β_3_ imaging. The probe molecules showed α_v_β_3_ expression in PC-3 cells in focal contacts on the cell surface, and also located intracellularly, possibly in cytosolic vesicles [239]. Given the number of small molecules reported as anti-integrin agents, there is potential for the rapid development of a large number of small molecule-based integrin imaging agents with wide ranging selectivities in future.

## 7. Conclusions and Future Prospects

RGD-binding integrins are a promising target for prostate cancer therapy in advanced disease. α_v_β_3_, α_v_β_5_ and α_5_β_1_ play key roles in mediating attraction and binding of tumour cells to the bone microenvironment. The presence of both β_3_ integrins in the microenvironment contributes to tumour progression through endothelial cell and osteoclast α_v_β_3_ supporting tumour angiogenesis and growth in bone and α_IIb_β_3_ supporting haematogenous metastasis. There is increasing evidence for involvement of α_v_β_6_ in tumour progression. Extensive co-operation and redundancy of function between RGD-binding integrins, and the identification of α_v_ as a node in the cell signalling network supporting advanced prostate cancer give a strong case for development of multi-integrin antagonists targeting the RGD-binding subfamily as molecularly targeted treatments for advanced and metastatic prostate cancer. The small molecule antagonist GLPG0876 is likely to provide partial proof of concept for this approach but its low affinity for α_IIb_β_3_ means it will not demonstrate the full potential. The number of selective integrin targeted agents already showing potential for tumour imaging and targeted therapy provides a supply of compounds for use in combination therapies, and templates for development of further compounds.
